# Chondroitin 6-Sulfation Regulates Perineuronal Net Formation by Controlling the Stability of Aggrecan

**DOI:** 10.1155/2016/1305801

**Published:** 2016-01-14

**Authors:** Shinji Miyata, Hiroshi Kitagawa

**Affiliations:** ^1^Institute for Advanced Research, Nagoya University, Furo-cho, Nagoya 464-8601, Japan; ^2^Bioscience and Biotechnology Center, Nagoya University, Furo-cho, Nagoya 464-8601, Japan; ^3^Department of Biochemistry, Kobe Pharmaceutical University, 4-19-1 Motoyamakita-machi, Kobe 658-8558, Japan

## Abstract

Perineuronal nets (PNNs) are lattice-like extracellular matrix structures composed of chondroitin sulfate proteoglycans (CSPGs). The appearance of PNNs parallels the decline of neural plasticity, and disruption of PNNs reactivates neural plasticity in the adult brain. We previously reported that sulfation patterns of chondroitin sulfate (CS) chains on CSPGs influenced the formation of PNNs and neural plasticity. However, the mechanism of PNN formation regulated by CS sulfation remains unknown. Here we found that overexpression of chondroitin 6-sulfotransferase-1 (C6ST-1), which catalyzes 6-sulfation of CS chains, selectively decreased aggrecan, a major CSPG in PNNs, in the aged brain without affecting other PNN components. Both diffuse and PNN-associated aggrecans were reduced by overexpression of C6ST-1. C6ST-1 increased 6-sulfation in both the repeating disaccharide region and linkage region of CS chains. Overexpression of 6-sulfation primarily impaired accumulation of aggrecan in PNNs, whereas condensation of other PNN components was not affected. Finally, we found that increased 6-sulfation accelerated proteolysis of aggrecan by a disintegrin and metalloproteinase domain with thrombospondin motif (ADAMTS) protease. Taken together, our results indicate that sulfation patterns of CS chains on aggrecan influenced the stability of the CSPG, thereby regulating formation of PNNs and neural plasticity.

## 1. Introduction

Chondroitin sulfate proteoglycans (CSPGs) consist of core proteins with one or more covalently attached chondroitin sulfate (CS) chains and are essential components of the brain extracellular matrix (ECM). During late postnatal development, CSPGs condense around subpopulations of neurons and form lattice-like ECM structures called perineuronal nets (PNNs) that surround synaptic contacts on the soma and dendrites [1, 2]. The appearance of PNNs coincides with the termination of the critical period during which neural circuits are highly plastic [3]. Enzymatic disruption of PNNs by chondroitinase ABC treatment reactivates neural plasticity in the adult cerebral cortex after the critical period has ended, suggesting that formation of PNNs restricts neural plasticity in the adult brain [3]. In several regions of the brain, including the cerebral cortex, PNNs are selectively formed around inhibitory interneurons expressing parvalbumin (PV-cells), which is implicated in many neural processes including regulation of the critical period plasticity [4].

PNNs can modify PV-cell function by facilitating the sequestration of secreted proteins at the neuronal surface via interactions with CS chains. Otx2 homeoprotein produced in the retina and choroid plexus is transported to PV-cells in the cerebral cortex, where it promotes maturation of PV-cells and controls the timing of the critical period [5, 6]. In addition, PNNs capture other secreted molecules, such as semaphorin3A and neuronal activity-regulated pentraxin, to regulate PV-cell function [7, 8]. CS chains in PNNs are required for localization of these molecules at the PV-cell surface [5, 7–9].

CSPGs belonging to the lectican family (aggrecan, versican, neurocan, and brevican) are major components of PNNs [1, 2]. Lectican family members share an amino-terminal hyaluronan binding domain and a carboxy-terminal tenascin-R binding domain [10]. In PNNs, lecticans bind to hyaluronan, which is tethered to the neuronal surface by transmembrane hyaluronan synthase, and this interaction is enhanced by link proteins [1, 11]. Multimeric forms of tenascin-R cross-link lecticans and stabilize the PNN structure [12].

Formation of PNNs is regulated by spatiotemporal expression of CSPG core proteins and link proteins [10]. In addition, dynamic changes in sulfation patterns of CS side chains are also observed during brain development [9, 13]. CS chains are linear polysaccharides composed of a repeating disaccharide unit consisting of uronic acid (UroA) and* N*-acetylgalactosamine (GalNAc). In the biosynthetic pathways, GalNAc residues of the repeating disaccharide units are sulfated by chondroitin 6-sulfotransferase-1 (C6ST-1) or chondroitin 4-sulfotransferase-1 (C4ST-1), thereby generating 6-sulfation or 4-sulfation, respectively [14, 15]. Subsequently, a small portion of 6- and 4-sulfation are further sulfated to form disulfated disaccharide units. 6-sulfation is abundant in the juvenile brain, whereas 4-sulfation is dominant in the adult brain [9, 13]. We previously reported that transgenic (TG) mice overexpressing C6ST-1 retained juvenile-like CS sulfation throughout life and showed impaired PNN formation [9]. As a result, C6ST-1 TG mice maintained juvenile-like plasticity in adulthood.

PNNs can be labeled with the broad marker* Wisteria floribunda *agglutinin (WFA) lectin. CS chains on aggrecan are proposed to be recognized by WFA because neurons from aggrecan-deficient mice lack staining for WFA [16]. We previously reported that C6ST-1 TG mice showed decreased WFA-positive conventional PNNs and ectopic appearance of 6-sulfation-enriched PNNs, which were stained by CS56 antibody but not by WFA lectin [9]. Incorporation of Otx2 into PV-cells was dependent on CS sulfation pattern of perineuronal nets: Otx2 accumulates in PV-cells surrounded by WFA-positive PNNs, but not observed in PV-cells surrounded by CS56-positive PNNs. Although these results imply the importance of sulfation patterns of CS chains, the mechanism by which the forced expression of 6-sulfation disrupts PNN formation in the adult brain remains unknown. Here we report that overexpression of C6ST-1 selectively decreases aggrecan in the aged brain and inhibits its accumulation into PNNs.

## 2. Materials and Methods

### 2.1. Animals

C6ST-1 TG mice were described previously [9]. Mice were housed under specific pathogen-free conditions in an environmentally controlled clean room at the Institute of Laboratory Animals, Kobe Pharmaceutical University. All experiments were conducted according to institutional ethics guidelines for animal experiments and safety guidelines for gene manipulation experiments.

### 2.2. Developmental Expression of PNN Components

Brains of wild-type (WT) or C6ST-1 TG mice at different postnatal ages were homogenized with a tight-fitting Potter glass homogenizer in HBSS buffer containing 1% Triton X-100 and protease inhibitor cocktail and incubated on ice for 60 min. After centrifugation at 15,000 rpm for 30 min at 4°C, protein concentrations of supernatants were determined using a BCA assay kit (Thermo). For chondroitinase digestion, the brain lysate (600 *μ*g as protein) was digested with 5 milliunits of chondroitinase ABC (Seikagaku Corp.) for 2 h at 37°C. Chondroitinase-digested lysate (40 *μ*g as protein) was separated by 5 or 10% acrylamide gel electrophoresis, transferred onto PVDF membranes (GE Healthcare), and incubated overnight at 4°C with the primary antibodies described in Table 1. Undigested lysate was used for detection by CS56 antibody. The blots were subsequently incubated with the appropriate HRP-labeled secondary antibodies for 1 h at room temperature and developed with the ECL detection system (GE Healthcare).

### 2.3. Sequential Extraction of CSPGs from Adult Mouse Brain

Fractionation of brain tissue was performed as described previously with minor modifications [11, 17]. Brains from 6-month-old adult WT or C6ST-1 TG mice were homogenized in PBS containing 2 mM EGTA, 2 mM phenylmethylsulfonyl fluoride, and protease inhibitor cocktail (buffer 1). The homogenate was centrifuged at 15,000 rpm for 20 min at 4°C. The supernatant (PBS extract) was collected, and the pellet was further extracted with buffer 2 (buffer 1 containing 0.5% Triton X-100), followed by extraction with buffer 3 (buffer 2 containing 6 M urea). To obtain total brain extract, brains were directly extracted with buffer 3. The 6 M urea and total extracts were dialyzed against PBS, and the protein content of the extracts was quantified by BCA assay kit. 20 *μ*g aliquots pretreated with chondroitinase ABC were subjected to Western blot analysis as described above.

### 2.4. Aggrecan Degradation Assay by ADAMTS-5

Whole brain lysate was prepared from 6-month-old adult WT or C6ST-1 TG mice as described above, except that protease inhibitors were omitted. Lysate was incubated with 0–500 nM recombinant human ADAMTS-5 (R&D Systems) in 50 mM Tris-HCl (pH 7.5), 100 mM NaCl, 5 mM CaCl_2_, and 0.05% Triton X-100 for 4 h at 37°C. Samples were further digested with chondroitinase ABC and subjected to Western blot analysis as described above.

### 2.5. Quantification of Proteins on Western Blot

**Figure 1:**
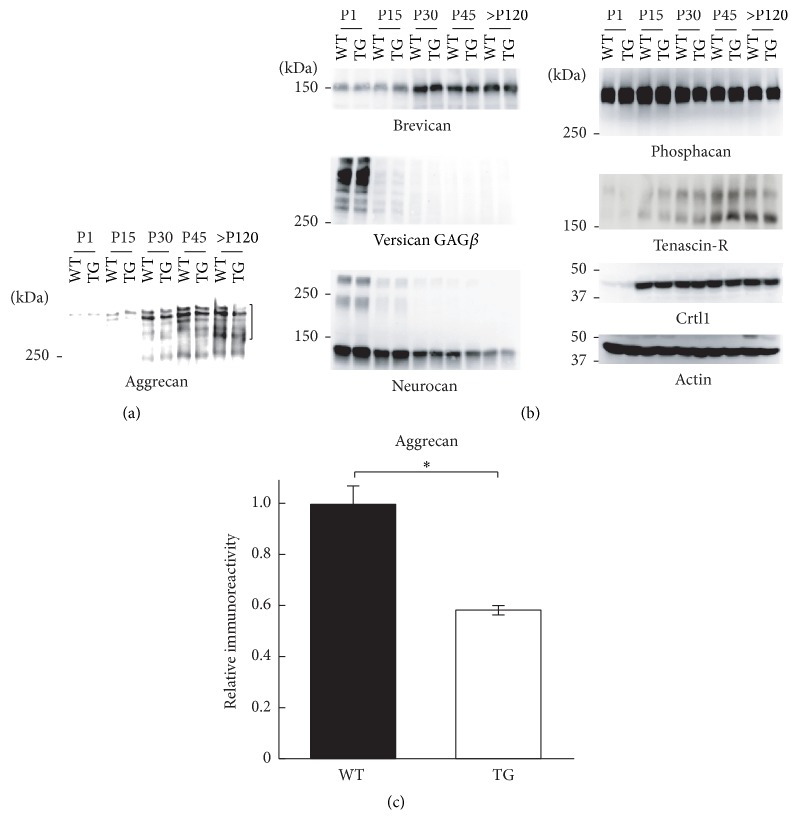
Developmental expression of PNN components in detergent-soluble fractions of WT and C6ST-1 TG mouse brain. (a) Expression of aggrecan in the brain of WT and C6ST-1 TG (TG) mice from postnatal day (P) 1 to >P120. (b) Expression of other PNN components, including brevican, versican GAG*β*, neurocan, phosphacan, tenascin-R, and Crtl-1, during development. Actin was detected as loading control. (c) Densitometric quantification of aggrecan in the aged brain (>P120). Intensity values were generated by measuring the signal intensity in the region indicated by the bracket. Asterisks denote significant differences (*P* < 0.05, Student's* t*-test) between WT and TG mice. Error bars represent SEM; *n* = 3 for each genotype.

**Figure 2:**
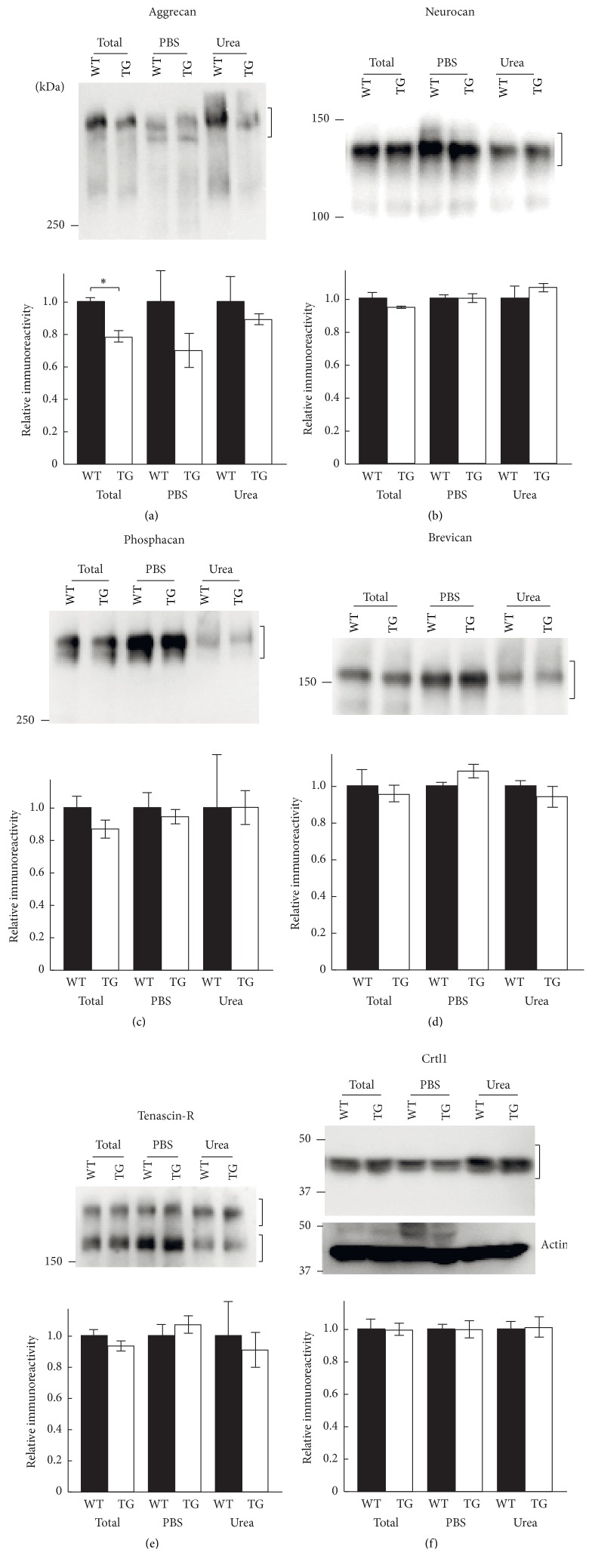
Sequential extraction of PNN components from adult mouse brain. Six-month-old adult WT and C6ST-1 TG mouse brain was sequentially extracted as described in Section 2. The same protein amount of the extracts was analyzed and intensities of the indicated bands were determined as relative values to WT mice for each extract. (a) The total level of aggrecan in C6ST-1 TG mice was significantly decreased compared with WT mice. Aggrecan levels in both the PBS extract and 6 M urea extract slightly reduced in C6ST-1 TG mice although this did not reach statistically significant difference. The levels of neurocan (b), phosphacan (c), brevican (d), tenascin-R (e), and Crtl1 (f) were not different between WT and C6ST-1 TG mice. Actin was detected as loading control. Asterisks denote significant differences (*P* < 0.05, Student's* t*-test) between WT and C6ST-1 TG mice. Error bars represent SEM; *n* = 3 for each genotype.

Intensity of the bands was determined using CS analyzer (ATTO) and the background was subtracted. For aggrecan, intensity was determined by measuring the signal intensity in the region indicated by the bracket in Figures 1 and 2.

### 2.6. Immunohistochemistry

Mice were perfused transcardially with PBS followed by 4% paraformaldehyde in PBS. Brains were removed and postfixed overnight with 4% paraformaldehyde in PBS. Coronal sections (40 *μ*m thick) were cut with a vibratome (DSK). Sections were permeabilized with 0.2% Triton X-100 in PBS, blocked with 2% BSA in PBS, and incubated overnight at room temperature with the primary antibodies described in Table 1. Sections were incubated with the appropriate Alexa488/594/647-labeled secondary antibodies (Invitrogen) for 1 h at room temperature. For WFA lectin staining, sections were incubated with biotinylated WFA followed by secondary labeling with Alexa594/647-conjugated streptavidin. Images were captured with an FV1200 laser scanning confocal microscope (OLYMPUS). For quantification of the number of WFA- and CS56-positive PNNs, labeled cells were counted in a 1.27 × 1.27 mm area spanning all cortical layers of the cerebral cortex. For three-dimensional reconstruction of PNNs, images were acquired at 0.5 *μ*m steps using a ×60 or ×100 objective and processed using FV10 ASW software (OLYMPUS).

### 2.7. Statistical Analysis

Statistical significance was determined using the unpaired two-tailed Student's* t*-test. Differences were considered significant at a* P* value of less than 0.05.

## 3. Results 

### 3.1. Developmental Expression of PNN Components in C6ST-1 TG Mice

We first examined developmental expression of CSPG core proteins, tenascin-R, and cartilage link protein 1 (Crtl1) in detergent-soluble fractions of the postnatal mouse brain (Figures 1(a) and 1(b)). Western blot analysis showed that expression of aggrecan, brevican, tenascin-R, and Crtl1 increased during postnatal development in both WT and C6ST-1 TG mice. In contrast, expression of neurocan and versican GAG*β* was high during early postnatal period and decreased with postnatal development. Phosphacan showed a peak of expression around postnatal day 15 and then decreased in the adult brain. Overall expression patterns of PNN components in C6ST-1 TG mice were similar to those of WT mice between postnatal days 1 and 45. However, we noticed that in the aged brain (>postnatal day 120) C6ST-1 TG mice showed decreased levels of aggrecan. Densitometric analysis revealed that the aggrecan level in C6ST-1 TG mice was significantly decreased compared with age-matched WT mice (Figure 1(c)).

To confirm the reduction of aggrecan in the aged C6ST-1 TG mice, we sequentially extracted PNN components with PBS and 6 M urea. It was reported that tightly associated components of PNNs were PBS-insoluble and can only be extracted in 6 M urea [17]. We found that the total level of aggrecan in the aged C6ST-1 TG mice was significantly decreased compared with controls (Figure 2(a)). In WT mice, aggrecan was much enriched in the 6 M urea-soluble fraction as compared to the PBS-soluble fraction. C6ST-1 TG mice showed slightly reduced aggrecan levels in both the PBS extract and 6 M urea extract, although it did not reach statistically significant difference. In contrast to aggrecan, other CSPGs, including brevican, neurocan, and phosphacan, were mostly extracted by PBS without 6 M urea (Figures 2(b)–2(d)). Considerable amounts of tenascin-R and Crtl1 were found in the 6 M urea-soluble fraction (Figures 2(e) and 2(f)). However, the levels of these molecules were not different between WT and C6ST-1 TG mice neither in the PBS extract nor 6 M urea extract.

### 3.2. Effects of Overexpression of C6ST-1 on the CS Moiety and the Oligosaccharide Linker to the Core Protein

**Figure 3:**
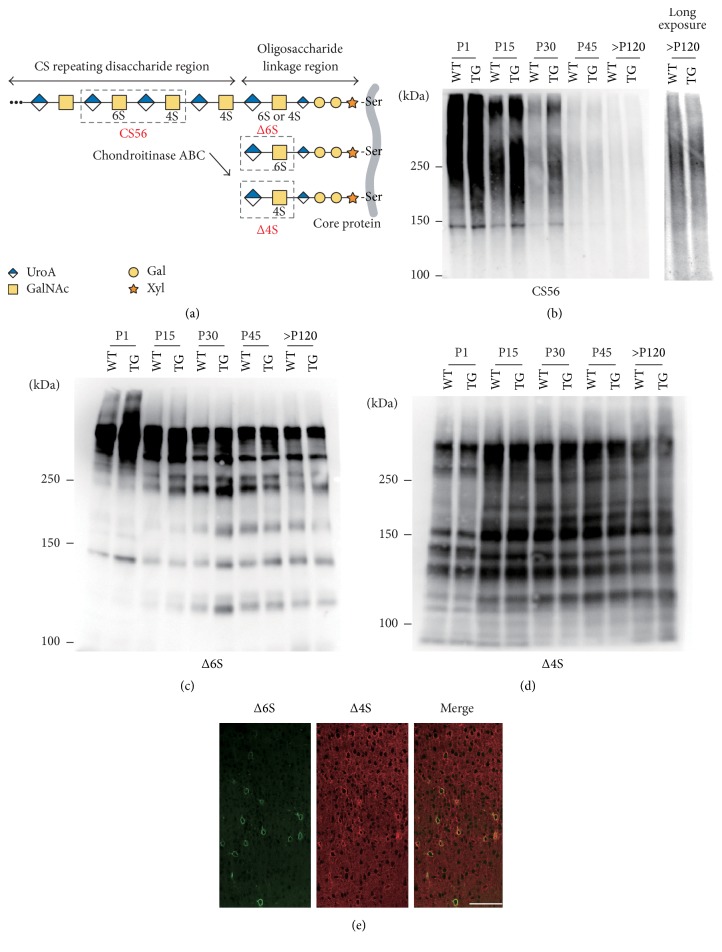
Effects of overexpression of C6ST-1 on the repeating disaccharide region and linkage region of CS chains. (a) Epitopes recognized by antibodies CS56, Δ6S, and Δ4S. 6S and 4S represent 6-sulfation and 4-sulfation, respectively. (b) C6ST-1 TG mice showed a delayed decrease in CS56-reactivity as compared with WT in the developing brain. Δ6S (c) and Δ4S (d) antibodies recognized distinct subsets of CSPGs. During early postnatal development, C6ST-1 TG mice exhibited greater Δ6S-reactivity than WT mice, whereas Δ4S-reactivity was not different between the two groups. (e) Distinct localization of Δ6S- and Δ4S-reactive CSPGs in 6-month-old adult cerebral cortex of WT mice. Scale bar, 100 *μ*m.

Overexpression of C6ST-1 may influence the sulfation pattern of the repeating disaccharide region and/or the linkage region of CS chains (Figure 3(a)). CS56 antibody was reported to recognize oligosaccharide structures containing 6-sulfation in the repeating disaccharide region of CS chains and its reactivity is lost after chondroitinase ABC digestion [9, 18]. In contrast, the so-called “anti-stub antibodies” recognize the linkage region oligosaccharide neoepitopes (Δ4S or Δ6S) that are generated after chondroitinase ABC treatment [19]. In WT mice, CS56-reactivity was abundant in neonatal brain and gradually decreased during postnatal development (Figure 3(b)). C6ST-1 TG mice showed higher CS56-reactivity than WT mice until postnatal day 30. However, CS56-reactivity was largely absent in the aged C6ST-1 TG mouse brain, proposing a possibility that CSPGs, which are abnormally modified by 6-sulfation, may be liable to degradation in the aged brain (see Section 3.4). Similar staining intensity was obtained from WT and C6ST-1 TG mice only after a long exposure time. Δ4S and Δ6S antibodies recognized distinct subsets of CSPGs, which showed differential developmental expression (Figures 3(c) and 3(d)). C6ST-1 TG mice showed slightly higher Δ6S-reactivity than WT mice, especially during early postnatal development (Figure 3(c)). In contrast, there was no substantial difference in Δ4S-reactivity between WT and C6ST-1 TG mice (Figure 3(d)). We also found that Δ6S-reactive CSPGs preferentially localized in PNNs, whereas Δ4S-reactive CSPGs were not limited to PNNs and were widely distributed throughout the cerebral cortex (Figure 3(e)), raising the possibility that localization of CSPGs into PNNs is regulated by the sulfation pattern of the CS chain linkage region. These results suggest that overexpression of C6ST-1 influences CS structures in both the repeating disaccharide and linkage region.

### 3.3. Condensation of Aggrecan Was Primarily Affected in C6ST-1 TG Mice

**Figure 4:**
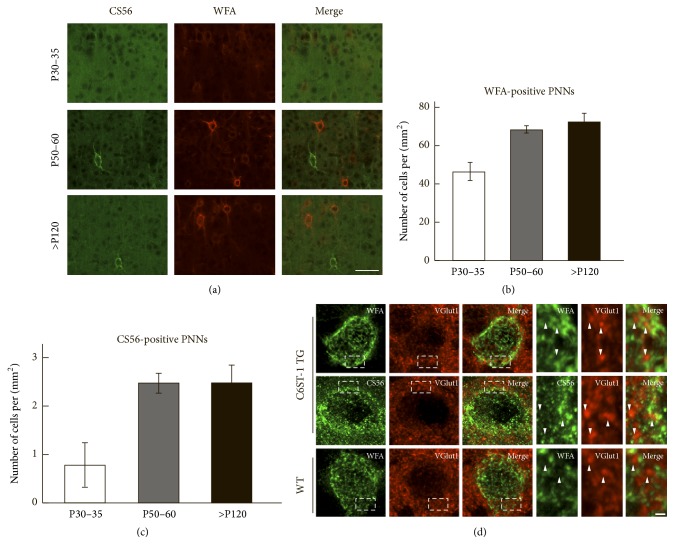
Developmental formation and morphological features of WFA-positive and CS56-positive PNNs. (a) Formation of WFA-positive and CS56-positive PNNs in the cerebral cortex of C6ST-1 TG mice during development. Note that CS56-positive PNNs were not colocalized with conventional WFA-positive PNNs. The numbers of both WFA-positive (b) and CS56-positive PNNs (c) increased until P60 and were maintained in aged C6ST-1 TG mice. Error bars represent SEM; *n* = 2-3. (d) Three-dimensional reconstruction revealed a distinct lattice-like structure of WFA-positive PNNs in both 6-month-old adult WT and C6ST-1 TG mice. In contrast, CS56-positive PNNs in C6ST-1 mice were sparse dot-like structures. Right: magnification of boxed regions in the left panels. VGluT1-labeled presynaptic terminals (arrowheads) were embedded in the meshwork of WFA-positive PNNs, whereas these terminals were not tightly surrounded by CS56-positive PNNs in C6ST-1 mice. Scale bars represent 50 *μ*m (a), 5 *μ*m ((d), left panels), and 1 *μ*m ((d), right panels).

We previously reported that, in the cerebral cortex of young adult C6ST-1 TG mice, a small portion of PV-cells was enclosed by 6-sulfation-enriched PNNs, which were labeled by the CS56 antibody, but not the conventional PNN marker WFA lectin (Figure 4(a)) [9]. We found that, during postnatal development of C6ST-1 TG mice, both WFA-positive and CS56-positive PNNs gradually increased until postnatal day 60 (Figures 4(a)–4(c)). Thereafter, the numbers of both PNNs were maintained in the aged animals (>postnatal day 120).

Three-dimensional reconstruction of WFA-positive PNNs displayed condensed lattice-like structures surrounding soma and proximal dendrites in both WT and C6ST-1 TG mice (Figure 4(d)). WFA-positive PNNs enwrapped presynaptic terminals labeled by antibody to vesicular glutamate transporter 1 (VGlut1). In contrast, CS56-positive PNNs in C6ST-1 TG mice showed sparse dot-like particles rather than a meshwork structure and did not tightly surround presynaptic terminals (Figure 4(d)).

**Figure 5:**
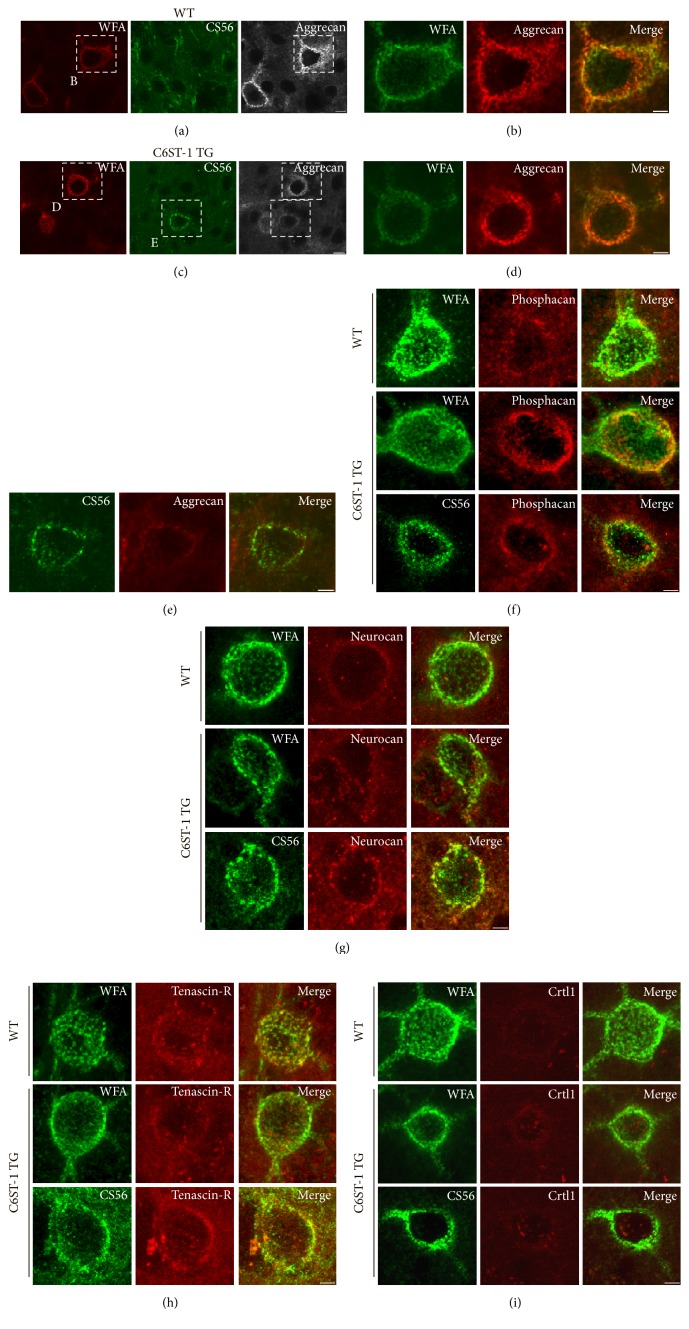
Impaired condensation of aggrecan into PNNs by overexpression of 6-sulfation. ((a), (b)) In 6-month-old adult WT mice, aggrecan showed a well-formed meshwork, which is similar to WFA lectin staining. (b) Magnification of boxed regions in (a). ((c)–(e)) In age-matched C6ST-1 TG mice, WFA-positive PNNs showed marked condensation of aggrecan, whereas neighboring CS56-positive PNNs showed diffuse and less condensed aggrecan staining. ((d), (e)) Magnification of boxed regions in (c). Immunolocalization of other PNN components including phosphacan (f), neurocan (g), tenascin-R (h), and Crtl1 (i) in WFA-positive and CS56-positive PNNs. In contrast to the impaired accumulation of aggrecan in CS56-positive PNNs in C6ST-1 TG mice, condensation of other PNN components was comparable between WT and C6ST-1 TG mice. Scale bars, 5 *μ*m.

We next examined which PNN components account for the difference between the two distinct PNNs. Staining with antibody recognizing core protein portion of aggrecan revealed a well-formed meshwork and a similar pattern to that with WFA lectin in WT mice (Figures 5(a) and 5(b)). As consistent with previous finding [9], CS56-positive PNNs were not observed even in the aged WT mice. In C6ST-1 TG mice, WFA-positive PNNs showed marked condensation of aggrecan core protein, which is similar to that observed in WT mice (Figures 5(c) and 5(d)). In contrast, aggrecan staining of neighboring CS56-positive PNNs appeared diffuse and less condensed over the soma (Figure 5(e)).

Neurocan and phosphacan were also observed in PNNs but did not show clear lattice-like structures. We observed no difference in staining of these CSPGs between WFA-positive and CS56-positive PNNs, suggesting that condensation of these CSPGs was not influenced by overexpression of 6-sulfation (Figures 5(f) and 5(g)). Brevican did not accumulate in PNNs neither in WT nor in C6ST-1 TG mice (data not shown). In addition, accumulation of tenascin-R and Crtl1 was not affected in CS56-positive PNNs compared with WFA-positive PNNs (Figures 5(h) and 5(i)). These results indicate that overexpression of 6-sulfation primarily impairs condensation of aggrecan into PNNs with little effect on other PNN components.

### 3.4. Overexpression of 6-Sulfation Rendered Aggrecan More Susceptible for Degradation by ADAMTS-5

**Figure 6:**
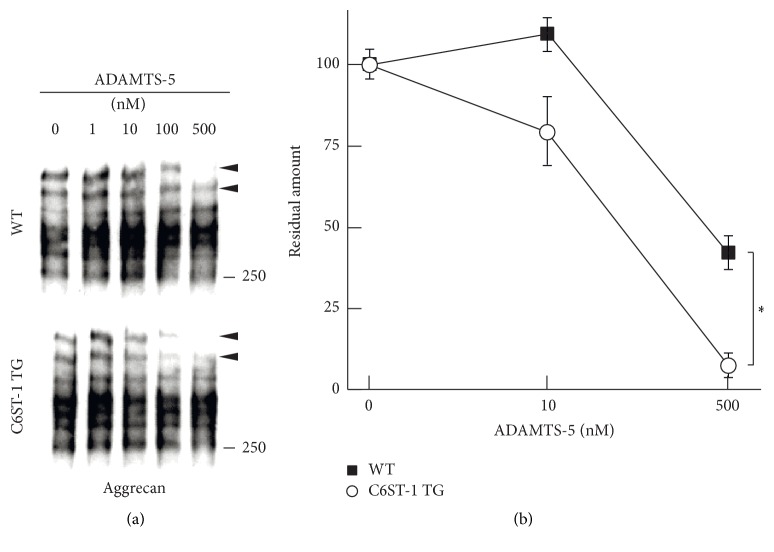
Degradation of aggrecan by ADAMTS-5* in vitro*. (a) Whole brain lysate of 6-month-old adult WT or C6ST-1 TG mice was digested with increasing amounts of ADAMTS-5. The concentrations of ADAMTS-5 used for digestion were indicated. Degradation of aggrecan was compared by Western blotting. Two high molecular weight aggrecan bands shown by arrowheads in C6ST-1 TG mice were degraded more efficiently by ADAMTS-5 than those of WT mice. (b) The residual amounts of two high molecular weight bands in WT (filled square) and C6ST-1 TG (open circle) mice were expressed as values relative to the no enzyme control. Asterisks denote significant differences (*P* < 0.05, Student's* t*-test) between WT and C6ST-1 TG mice. Data ± SEM were obtained from the triplicate experiments.

Finally, we examined whether aggrecan produced in C6ST-1 TG mice is liable to degradation by ADAMTS-5 (aggrecanase-2), which has been proposed as an aggrecan-degrading enzyme and is expressed in the adult mouse brain [20, 21]. Whole brain lysate was digested with various concentration of recombinant ADAMTS-5 and degradation of aggrecan was compared by Western blotting. We found that high molecular weight aggrecan bands in C6ST-1 TG mice were degraded more efficiently by ADAMTS-5 than those of WT mice (Figures 6(a) and 6(b)), indicating that increased 6-sulfation accelerated proteolysis of aggrecan by ADAMTS-5.

## 4. Discussion

Our study revealed that overexpression of 6-sulfation markedly decreased aggrecan in the aged brain. Previous studies have implicated the involvement of CS chains in the metabolism of aggrecan in cartilage. Mice deficient in chondroitin GalNAc transferase-1 (ChGn-1), which catalyzes the initial step of CS biosynthesis, show a decreased amount of CS chains and an accelerated degradation of aggrecan in the cartilage [22, 23]. Together with our data, this indicates that proper sulfation patterns of CS chains play an essential role in the stability of aggrecan, probably by providing protection from aggrecan-degrading enzymes.

Several members of metalloproteinases family and ADAMTS family cleave CSPGs including aggrecan in the ECM [20]. ADAMTS-4 (aggrecanase-1) and ADAMTS-5 (aggrecanase-2) have been proposed to be responsible for aggrecan degradation [20]. However, recent study using ADAMTS-4 and ADAMTS-5 knockout mice suggested the presence of additional aggrecan-degrading enzymes in the spinal cord [24]. In the cerebral cortex, ADAMTS-8 and ADAMTS-15 are exclusively expressed by PV-cells, which are surrounded by PNNs [25, 26], suggesting that these may be novel aggrecan-degrading enzymes specifically involved in turnover and remodeling of PNNs* in vivo*. Our results imply that juvenile-type CS sulfation rich in 6-sulfation allows remodeling of PNNs by ADAMTSs, thereby keeping high plasticity during the critical period. The developmental shift of sulfation patterns from 6-sulfation to 4-sulfation may render aggrecan resistant to degradation and stabilize PNNs in adult brain. Some ADAMTSs have been shown to cleave CSPGs in a manner depending on CS chains attached to core proteins [27, 28]. Thus, further studies are needed to determine how CS sulfation patterns on aggrecan are involved in CSPG degradation by ADAMTSs. It is currently unknown why aggrecan is selectively decreased by excess 6-sulfation. Aggrecan is distinctive from other CSPGs in terms of the number of CS chains attached to the core protein, because it contains approximately one hundred CS chains per core protein [29]. Thus aggrecan may be highly susceptible to structural changes in CS chains.

In spite of the disturbed condensation of aggrecan into PNNs in C6ST-1 TG mice, other PNN components appeared to be unaffected, indicating that these components accumulate into PNNs in an aggrecan-independent manner. This is consistent with previous reports in which condensation of Crtl1, tenascin-R, and brevican is unaffected in neuronal cultures prepared from aggrecan-deficient mice [16, 30]. Production of aggrecan and formation of WFA-positive PNNs are dependent on neuronal activity, whereas tenascin-R and brevican are produced in a glia-dependent manner [31–33]. Thus, there may be at least two independent mechanisms of PNN formation: one is neuron-dependent and the other is glia-dependent. However, the contribution of the glia-dependent mechanism seems to be unnecessary for the development of PNNs, because previous reports found that WFA-positive PNNs were formed in dissociated neuronal culture in the absence of glial cells [31, 34]. In addition, we previously demonstrated* in vivo *that PNN formation and Otx2 accumulation were locally manipulated by PV-cell autonomous production of 6-sulfation [9]. These results indicate that CSPGs produced by neurons, most likely aggrecan, are required for the proper function of PNNs.

In this study we found that overexpression of C6ST-1 increases 6-sulfation in the repeating disaccharide region as well as the linkage region of CS chains. Chondroitinase ABC treatment releases Otx2 and semaphorin3A from PNNs, indicating that these molecules bind to the repeating disaccharide region of CS chains [7, 35]. Indeed, it was reported that both Otx2 and semaphorin3A selectively interact with CS chains rich in disulfated E units [GlcA-GalNAc(4,6-*O*-disulfate)] [35–37]. However, the significance of the linkage region of CS chains in PNN formation is unknown. Here we show differential localization of Δ4S- and Δ6S-reactive CSPGs, proposing the possibility that sulfation of the linkage region influences localization of CSPGs into PNNs. Our laboratory previously demonstrated that 4-sulfation of the GalNAc residue in the linkage region catalyzed by C4ST-2 triggers the elongation of chondroitin backbone, which is prerequisite for the formation of sulfation patterns of the repeating disaccharide units [38, 39]. Therefore, it is possible that sulfation of the linkage region affects assembly of CSPGs within the ECM by modulating sulfation patterns of the repeating disaccharide region.

WFA lectin is widely used to label PNNs. However, a small portion of PNNs in C6ST-1 TG mice was devoid of WFA-labeling but was labeled by CS56 antibody recognizing oligosaccharide structures containing 6-sulfation. It has been proposed that WFA recognizes a CS structure on aggrecan because WFA-staining is abolished by either chondroitinase ABC digestion of CS chains or deletion of aggrecan [3, 16]. However, the precise structure recognized by WFA is unknown. During formation of PNNs, there is a progressive decrease in 6-sulfation that is mirrored by an increase in 4-sulfation [9, 13]. Furthermore, C6ST-1 TG mice contain less 4-sulfation and more 6-sulfation than WT mice, which is accompanied by decreased WFA-staining and increased CS56-staining. Thus, WFA may recognize a CS structure on aggrecan consisting of 4-sulfation. Notably, drastic changes in WFA-reactivity have been reported in the brains of patients with schizophrenia. In schizophrenic brain, the number of WFA-positive PNNs around PV-cells is markedly decreased [40–42]. In addition, WFA-labeled astrocytes are increased, whereas CS56-labeled astrocytes are decreased in schizophrenia [43]. Taken together, an abnormal balance of 4-sulfation and 6-sulfation produced by both neurons and astrocytes may contribute to the disease.

## Figures and Tables

**Table 1:** List of the antibodies used in this study.

Antigen	Isotype	Source	Dilution
Aggrecan	Rabbit IgG	Millipore, AB1031	1 : 2000 for WB, 1 : 200 for IHC
Brevican	Mouse IgG1	BD Transduction Laboratories, 610894	1 : 2000 for WB, 1 : 200 for IHC
Versican GAG*β*	Rabbit IgG	Millipore, AB1032	1 : 2000 for WB
Neurocan	Sheep IgG	R&D Systems, AF5800	1 : 3000 for WB, 1 : 200 for IHC
Phosphacan	Mouse IgG1	DSHB, 3F8	1 : 200 for WB, 1 : 5 for IHC
Tenascin-R	Mouse IgG1	R&D Systems, MAB1642 (clone 619)	1 : 2000 for WB, 1 : 200 for IHC
Crtl1	Goat IgG	R&D Systems, AF2608	1 : 2000 for WB, 1 : 200 for IHC
CS56	Mouse IgM	Sigma, C8035 (clone cs-56)	1 : 2000 for WB, 1 : 200 for IHC
Δ6S	Mouse IgM	Cosmo Bio, CAC-PRPG-BC-M04 (clone 3B3)	1 : 200 for WB, 1 : 50 for IHC
Δ4S	Mouse IgG1	Millipore, MAB2030 (clone BE-123)	1 : 20000 for WB, 1 : 1000 for IHC
VGlut1	Guinea pig IgG	Millipore, AB5905	1 : 1000 for IHC
WFA	Lectin	EY Laboratories, BA-3101-1	1 : 1000 for IHC

WB, Western blot. IHC, immunohistochemistry.
